# Achieving Accurate Ligament Balancing Using Robotic-Assisted Unicompartmental Knee Arthroplasty

**DOI:** 10.1155/2013/837167

**Published:** 2013-03-24

**Authors:** Johannes F. Plate, Ali Mofidi, Sandeep Mannava, Beth P. Smith, Jason E. Lang, Gary G. Poehling, Michael A. Conditt, Riyaz H. Jinnah

**Affiliations:** ^1^Department of Orthopaedic Surgery, Wake Forest School of Medicine, Medical Center Boulevard, Winston-Salem, NC 27157-1070, USA; ^2^Morriston Hospital, Swansea SA6-6NL, UK; ^3^MAKO Surgical Corp., 2555 Davie Road, Fort Lauderdale, FL 33317, USA

## Abstract

Unicompartmental knee arthroplasty (UKA) allows replacement of a single compartment in patients with limited disease. However, UKA is technically challenging and relies on accurate component positioning and restoration of natural knee kinematics. This study examined the accuracy of dynamic, real-time ligament balancing using a robotic-assisted UKA system. Surgical data obtained from the computer system were prospectively collected from 51 patients (52 knees) undergoing robotic-assisted medial UKA by a single surgeon. Dynamic ligament balancing of the knee was obtained under valgus stress prior to component implantation and then compared to final ligament balance with the components in place. Ligament balancing was accurate up to 0.53 mm compared to the preoperative plan, with 83% of cases within 1 mm at 0°, 30°, 60°, 90°, and 110° of flexion. Ligamentous laxity of 1.31 ± 0.13 mm at 30° of flexion was corrected successfully to 0.78 ± 0.17 mm (*P* < 0.05). Robotic-assisted UKA allows accurate and precise reproduction of a surgical balance plan using dynamic, real-time soft-tissue balancing to help restore natural knee kinematics, potentially improving implant survival and functional outcomes.

## 1. Introduction 

Unicompartmental knee arthroplasty (UKA) has seen resurgence in the past decade with approximately 51,300 cases performed in 2009 and an estimated growth of 32.5% annually [1–3]. Benefits of UKA compared to total knee arthroplasty include reduced blood loss, reduced perioperative morbidity, faster recovery, shorter rehabilitation, increased postoperative range of motion, and reduced surgical cost [4–9]. However, proper patient selection is vital and the procedure remains technically demanding as the minimally invasive procedure limits surgical exposure and impedes precise component alignment and fixation [3, 6, 10–14]. UKA failures have mainly been attributed to improper component alignment leading to altered knee biomechanics with accelerated polyethylene wear if deformity is undercorrected, disease progression in other compartments if overcorrected, and anterior knee pain [6, 8, 15–17]. UKA component position and alignment are intricately associated with soft-tissue balancing during this procedure.

UKA allows for minimal disruption of the patient's native anatomy and is intended to restore the normal height of the affected compartment to produce normal ligament tension during the flexion-extension cycle. The success of UKA relies on proper soft-tissue tensioning to obtain a balanced flexion-extension gap and varus-valgus stability [14]. While advances in surgical instrumentation with improved alignment guides and cutting blocks for minimally invasive surgery and navigation systems have improved component positioning in UKA, soft-tissue tensioning is still dependent on surgeon ability and experience. Achieving proper ligament balance throughout the flexion-extension cycle and avoiding tightness or laxity are complex and partly rely on component size and position [14, 18]. Increased soft-tissue tightness may decrease the range of motion and increase wear while increased laxity may lead to joint instability and knee pain. 

Robotic-assisted UKA allows for improved component positioning [2, 3, 19] with the ability of real-time, dynamic ligament balancing intraoperatively. The robotic system uses optical motion capture technology that dynamically tracks intracortical markers fixed to the tibia and femur. The purpose of the current study was to describe the technique of soft-tissue tensioning and assess the accuracy of robotic-assisted ligament balancing based on an intraoperative balance plan during 52 consecutive medial robotic-assisted medial UKAs. We hypothesized that robotic-assisted UKA accurately produces ligament tension according to an intraoperative balance plan devised before component implantation. 

## 2. Material and Methods

### 2.1. Robotic-Assisted Ligament Balancing Technique for UKA

**Figure 1:**
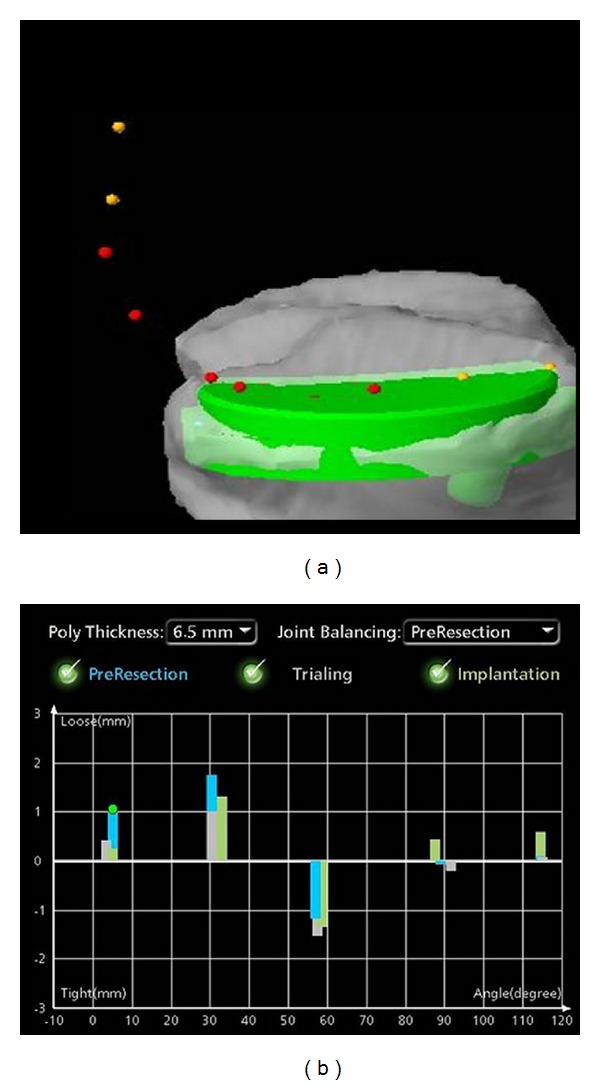
Ligament balancing was measured throughout various angles during the flexion-extension cycle relative to tibia and mechanical axis. (a) The colored dots represent measurements during femoral range of motion. (b) Intraoperative screenshot of the robotic system showing ligament balance at 0°, 30°, 60°, 90°, and 110° of flexion before resection, with the trial component in place, and after implantation.

While the surgical technique using a robotic-assisted UKA system has been described elsewhere in detail [6], this paper will focus on how to obtain accurate ligament balance for replacement of the medial compartment. Preoperative CT scans are used by the computer system to render a three-dimensional model of patient anatomy. Intraoperatively, anatomic landmarks are used to register the patient to the robot following intracortical placement of the femoral and tibial marker array. A minimally invasive medial joint incision is made, and medial osteophytes are resected. The knee is then ranged through a number of flexion-extension cycles. Valgus stress is then applied by the surgeon to open up the medial compartment and bring the knee into its “natural” alignment. The ligament balance is then analyzed and displayed by the computer system in real time as deviation from the optimal tracking pattern of the prosthesis calculated by the computer in millimeters (mm) during numerous flexion-extension cycles at 0°, 30°, 60°, 90°, and 110° of flexion (Figure 1). Negative deviation depicts ligamentous tightness and positive values indicate ligamentous laxity.

The values obtained during the range of motion with valgus stress serve as the intraoperative balance plan for ligamentous tensioning. Using the computer system, component position or size can be altered, and the resulting changes in predicted ligament balance can be observed in real-time. If there is predicted laxity, component size and position can be changed to increase tightness, thereby programming the robot to alter bone cuts based on the preoperative CT scans and intraoperative findings. After the bone resections have been made using the robotic arm, the trial components are inserted and ligamentous tension is compared to the intraoperative balance plan. If proper balance is achieved with the trial components in place, the final components are inserted and cemented, and final ligamentous balance is obtained during range of motion.

### 2.2. Assessment of Ligament Balancing Accuracy

The intraoperative data from 51 consecutive patients (52 knees) who underwent robotic-assisted UKA (MAKOplasty, MAKO Surgical Corp.) of the medial compartment by a single surgeon (RHJ) were prospectively collected over a 6-month period. All patients received a fixed-bearing UKA with an onlay cemented tibial component and cemented femoral component. Following registration of the robotic system and prior to incision, the intraoperative balance plan for ligament tensioning was obtained under valgus stress. After implantation of the final components, dynamic measurements were repeated without valgus stress. Data was stored on the computer system (Figure 1), and the actual ligament balancing was compared to the intraoperative balance plan by subtracting the planned measurements at 0°, 30°, 60°, and 90° of flexion from the actual postoperative measurements. Analysis of variance (ANOVA) was used to compare ligament balance at 0°, 30°, 60°, 90°, and 110° of flexion with Bonferroni post-hoc comparison with alpha 0.05. All data are presented as mean ± standard error of the mean (SEM). 

## 3. Results

**Figure 2:**
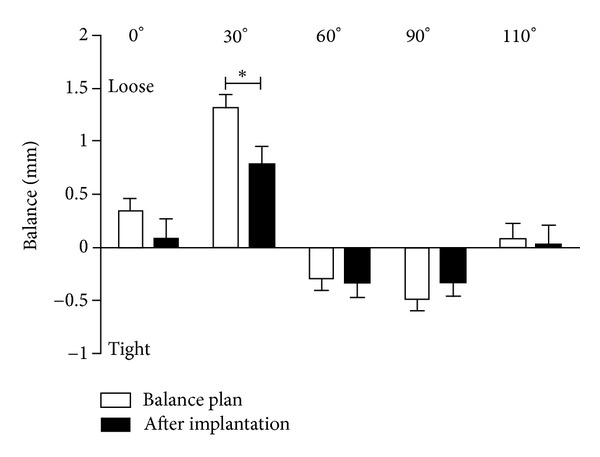
Analysis of ligament balance at various degrees of knee flexion. The intraoperative balance plan was similar measurements obtained after component implantation at 0°, 60°, 90°, and 110°. At 30°, ligament balance was relatively loose and surgically corrected, revealing a significant difference (*P < 0.05) between the balance plan and measurements after component implantation.

The mean age of patients in this study was 67 years (range, 50–90 years) with a mean body mass index of 31.4 kg/m^2^ (range, 21.5–43.8 kg/m^2^). The surgical indication in all patients was isolated osteoarthritis of the medial compartment of the knee. Intraoperative measurements under valgus stress before component implantation revealed that ligamentous balance significantly changed during the flexion-extension cycle (Figure 2, *P* < 0.001). At 0° (0.34 ± 0.12 mm) and 30° (1.31 ± 0.13 mm) of flexion, the ligaments were relatively loose, at 60° (−0.28 ± 0.11 mm) and 90° (−0.49 ± 0.12 mm) of knee flexion ligaments were relatively tight, and at 110° of flexion close to neutral (0.07 ± 0.15). Comparison of the intraoperative balance plan to measurements after component implantation revealed similar ligament balance at 0° (0.11 ± 0.17 mm), 60° (0.78 ± 0.18 mm), 90° (−0.28 ± 0.13 mm), and 110° (−0.02 ± 0.19 mm) degrees of flexion (*P* > 0.05). Ligament balance at 30° of flexion was significantly reduced (0.88 ± 0.18 mm) after component implantation compared to the intraoperative balance plan indicating tighter ligament balance (*P* < 0.05). 

**Figure 3:**
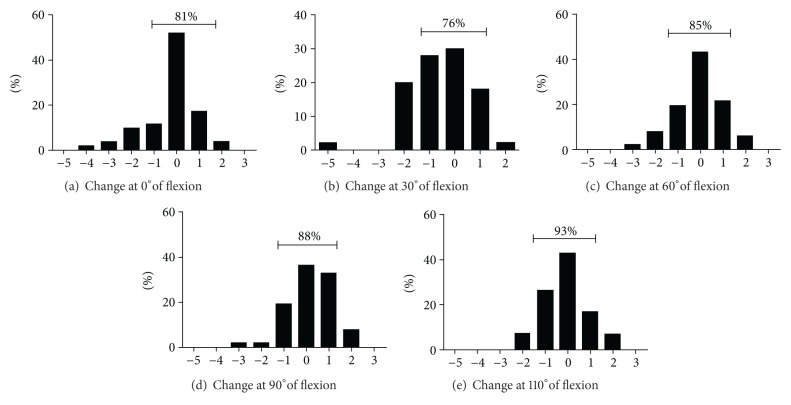
At 0° (a), 60° (c), 90° (d), and 110° (e), ligament balance between 1 mm and −1 mm was achieved in 81% to 93% of cases. At 30° (b), 76% of cases were balanced between 1 mm and −1 mm due to a necessary increase in ligament tightness.

Overall, the variation in ligament tensioning between the intraoperative balance plan and measurements after component implantation was less than 1 mm in 83% of the cases (Table 1 and Figure 3). At 0°, the mean change was −0.26 ± 0.17 mm (range, −4.40–2.20 mm), at 30°  −0.53 ± 0.18 mm (range, −5.30–1.80 mm), at 60°  −0.04 ± 0.15 mm (range, −3.10–2.30 mm), at 90°  0.16 ± 0.13 mm (range, −2.70–2.00 mm), and at 110°  −0.10 ± 0.14 mm (range, −2.2–2.0). 

## 4. Discussion and Conclusion

Successful outcomes of UKA rely on the restoration of normal knee kinematics and muscle lever arms of the knee joint. Therefore, restoration of proper ligamentous length and tension is a vital component of the UKA surgical technique. Using a robotic-assisted UKA system, we showed that real-time, dynamic ligament balancing reproduced planned ligamentous balance and, when appropriate, was able to increase ligament tightness when there was relative preoperative laxity. 

Whiteside pointed out that proper ligament balance in combination with component alignment and fixation is vital for the success of UKA [14]. In a normal knee, the ligaments and menisci control anterior-posterior and varus-valgus movement between the femur and tibia. Medial compartment osteoarthritis with loss of cartilage and bone substance leads to a varus deformity and contracture of the medial capsule and ligaments [20]. The goal of medial UKA for a correctable varus deformity is to restore the normal height of the compartment, thereby achieving ligamentous balance and natural alignment of the joint. This “gap filling” procedure is in contrast to total knee arthroplasty in which bone cuts are made first and then soft-tissues are released to obtain a rectangular flexion-extension gap. Component malpositioning by only 2° during UKA can lead to failure [6, 9, 10, 13, 21], because normal joint biomechanics are altered without achieving proper ligamentous balance possibly leading to increased polyethylene wear and accelerated progression of degenerative disease in the uninvolved compartment [15–17].

During conventional UKA, soft-tissue balance is assessed with the trial components in place and with subjective varus-valgus stress testing, commonly at 0° and 90° [22]. The restoration of the normal height of the compartment is vital to achieve proper ligamentous balance. The appropriate ligament balance is left to surgeon's feel and has been described as an art that requires ability and experience [23]. While intraoperative measuring devices are available for total knee arthroplasty [23], their use remains ambiguous and there is currently no such device available for UKA. Navigation systems for UKA have become available to improve component positioning and alignment; however, these systems are incapable of assessing ligament balance. Robotic-assisted systems assess ligamentous balance dynamically and in real-time at various flexion angles. Placing a valgus stress on the knee after medial osteophytes have been removed opens the medial compartment and brings the knee into its natural alignment. These measurements enable the fine tuning of the planned component position to achieve optimal component height and orientation, and thereby ligamentous balance. Following bone resection using the high speed burr with haptic feedback, the femoral and tibial trial components are inserted, and balance measurements are repeated. If necessary, bone cuts can be adjusted for optimal implant orientation. Using a robotic-assisted UKA system, the surgeon has the ability to measure ligament tightness or laxity objectively during dynamic, real-time analysis by the computer system. Natural knee kinematics can be restored based on objective measurements, in addition to surgical acumen. 

Specifically, fixed-bearing tibial components, such as the implants used in this study, rely on proper soft-tissue tensioning. There is low conformity between the femoral and tibial components with low contact areas allowing for unconstrained movements between the femur and tibia controlled only by the ligamentous apparatus [24]. Conversely, mobile-bearing UKA systems have high conformity of the tibial and femoral components to increase their contact areas and reduce contact stress. Mobile-bearing systems came in favor to reduce contact stress of the articulating surface thereby preventing polyethylene fatigue and failure [24]. With highly-crosslinked polyethylene components available that are more resistant to wear, Burton et al. and Taddei et al. showed decreased wear during *in vitro* testing with a fixed-bearing UKA compared to a mobile-bearing UKA [24, 25]. In a recent meta-analysis of clinical, radiological, and kinematic outcomes comparing fixed- to mobile-bearing UKA, Smith et al. showed similar improvements and outcomes between 146 mobile-bearing UKAs and 147 fixed-bearing UKAs at a mean 5.8 ± 3.1 years [26]. Despite the design of the prosthesis, proper ligament balance is essential for long-term survival and functional improvements.

There have been numerous advances in UKA instrumentation and cement or cementless fixation techniques that have led to an increase in the survivorship of UKA in the past decade [27, 28]. Minimal invasive instrumentation has become available for more precise component positioning, and improvements in polyethylene components have led to decreased wear. However, robotic-assisted UKA systems have been shown to increase the precision of component placement [2, 19, 29], and the opportunity for real-time, dynamic ligament balancing offers an additional advantage. Dunbar et al. assessed the accuracy of component placement in 20 patients who received postoperative CT scans [29]. In comparison to the preoperative plan, accuracy (root-mean-square error) for femoral and tibial component placement was within 1.6 mm and 3.0° in all directions [29]. Lonner et al. compared tibial component alignments between manual UKA and robotic-assisted UKA and found a greater variance in component position, increased tibial slope, and increased varus alignment when the tibia was prepared manually [19]. 

A major limitation of this study is the lack of clinical or functional outcomes in this patient cohort; the study was intended to assess the accuracy of ligament tensioning only based upon the intraoperative balance plan. There are currently no studies available on the clinical outcomes of robotic-assisted UKA due to the novelty of the device. Certainly, long-term studies on the outcomes of the robotic-assisted device compared to manual UKA are needed to delineate a possible advantage of the robot in light of the financial investment. However, based on the technical demands of UKA, we believe that improved component positioning and alignment in combination with dynamic, real-time assessment of ligament balance offered by the robotic-assisted system may improve outcomes.

To our knowledge, this is the first study assessing real-time dynamic ligament balancing with a robotic-assisted system for UKA. We conclude from our findings that robotic-assisted UKA can accurately and precisely reproduce intraoperatively planned ligamentous balance using real-time, dynamic measurements. In combination with high accuracy of component placement, robotic-assisted systems may improve functional outcomes and survivorship of UKA patients; however, further investigations into the benefits of robotic systems for UKA are needed.

## Figures and Tables

**Table 1:** Comparison of the intra-operative balance plan and ligament balance measurements following component implantation. Data is expressed as mean ± standard error of the mean in millimeters.

Flexion angle	Balance plan	After implantation	Change in balance	*P* value
0°	0.34 ± 0.12	0.08 ± 0.18	−0.26 ± 0.17	*P* > 0.05
30°	1.31 ± 0.13	0.78 ± 0.17	−0.53 ± 0.18	*P* < 0.05*
60°	−0.28 ± 0.11	−0.33 ± 0.14	−0.04 ± 0.15	*P* > 0.05
90°	−0.49 ± 0.12	−0.32 ± 0.13	0.16 ± 0.13	*P* > 0.05
110°	0.03 ± 0.16	−0.07 ± 0.19	−0.10 ± 0.14	*P* > 0.05

*A *P* value less than 0.05 was considered statistically significant.
